# Association between Dietary Patterns and Depressive Symptoms Over Time: A 10-Year Follow-Up Study of the GAZEL Cohort

**DOI:** 10.1371/journal.pone.0051593

**Published:** 2012-12-12

**Authors:** Agnès Le Port, Alice Gueguen, Emmanuelle Kesse-Guyot, Maria Melchior, Cédric Lemogne, Hermann Nabi, Marcel Goldberg, Marie Zins, Sébastien Czernichow

**Affiliations:** 1 INSERM U1018, University of Versailles St Quentin. Centre for research in Epidemiology and Population Health, Villejuif, France; 2 UMR, INSERM U557, INRA U1125, Conservatoire National des Arts et Métiers, University of Paris XIII, Bobigny, France; 3 APHP, Hôpitaux Universitaires Paris Ouest, Service universitaire de Psychiatrie de l’adulte et du sujet âgé, Paris, France; 4 Université Paris Descartes, Sorbonne Paris Cité, Faculté de Médecine, Paris, France; 5 APHP, Ambroise Paré Hospital, Department of Nutrition, Boulogne-Billancourt, France; Brigham and Women’s Hospital and Harvard Medical School, United States of America

## Abstract

**Background:**

Data on the association between dietary patterns and depression are scarce. The objective of this study was to examine the longitudinal association between dietary patterns and depressive symptoms assessed repeatedly over 10 years in the French occupational GAZEL cohort.

**Methods:**

A total of 9,272 men and 3,132 women, aged 45–60 years in 1998, completed a 35-item Food Frequency Questionnaire (FFQ) at baseline. Dietary patterns were derived by Principal Component Analysis. Depressive symptoms were assessed by the Center for Epidemiologic Studies Depression scale (CES-D) in 1999, 2002, 2005 and 2008. The main outcome measure was the repeated measures of CES-D. Longitudinal analyses were performed with logistic regression based on generalized estimating equations.

**Principal Findings:**

The highest quartile of low-fat, western, high snack and high fat-sweet diets in men and low-fat and high snack diets in women were associated with higher likelihood of depressive symptoms at the start of the follow-up compared to the lowest quartile (OR between 1.16 and 1.50). Conversely, the highest quartile of traditional diet (characterized by fish and fruit consumption) was associated with a lower likelihood of depressive symptoms in women compared to the lowest quartile, with OR = 0.63 [95%CI, 0.50 to 0.80], as the healthy pattern (characterized by vegetables consumption) with OR = 0.72 [95%CI, 0.63 to 0.83] and OR = 0.75 [95%CI, 0.61 to 0.93] in men and women, respectively. However, there was probably a reverse causality effect for the healthy pattern.

**Conclusion:**

This longitudinal study shows that several dietary patterns are associated with depressive symptoms and these associations track over time.

## Introduction

Mood disorders, and in particular depression, are frequent across the globe. The lifetime prevalence of depressive disorders is estimated between 10% to 20% in Europe and North America, and is two times higher in women than in men [1]. In France, the lifetime prevalence of depression is estimated at 13–17% in men and 25–30% in women [2]. According to the World Health Organization, depression was the 4th cause of disability in the world in 2000 and will be the second cause in 2020 [3].

Diet is related to inflammation, oxidative stress and brain plasticity and function; all of these physiological factors are potentially involved in depression [4]. A recent review showed discordant results according to studies on the association between poly-unsaturated fatty acids (PUFA), fish, folate and other B vitamins and depressive symptoms [5], [6]. However, because nutrients are consumed in a combined way, the investigation of dietary patterns with a global perspective has been suggested as a more comprehensive approach than the study of specific nutrients. The effect of the overall diet may be easier to detect than the effects of individual dietary components, especially when the latter are of minor extent or when the global effect is greater than the sum of the parts. Using this approach, cross-sectional studies in Japan, Norway and Australia have shown a relationship between dietary patterns and depression in adults [4], [7], [8]. Subjects who have a healthy diet, defined as a high consumption frequency of fish, fruits and vegetables have a lower probability of depressive symptoms, while a high consumption of refined/processed foods is associated with increased risk of depressive symptoms. In the UK, a prospective study using a single measure of the Center for Epidemiologic Studies Depression scale (CES-D) demonstrated an increased likelihood of depression after 5 years for subjects adhering to a processed food dietary pattern and a reduced likelihood for subjects following a whole foods diet pattern (vegetables, fruits, fish) [7]. An increased probability of depression has been described among Chinese adolescent with high snacking patterns [9], while in Australia the same observation was described in cross-sectional and prospective analysis among adolescents consuming a western diet [10], [11]. In the only prospective study on middle-aged adults available in the literature, depressive symptoms were assessed at one point in time [7]. Others studies using a-posteriori methods to assess dietary patterns, i.e. scores of existing and well know diets, as the Mediterranean diet, have shown relation between high score of consumption and decreased symptoms of depression [12]. Thus, it remains unclear whether the relationship between dietary patterns and depression symptoms tracks over time. Our aim was to study the longitudinal association between dietary patterns and depressive symptoms assessed four times, among public-sector employees in France who participated in the GAZEL cohort study between 1998 and 2008.

## Subjects and Methods

### Subjects

The GAZEL cohort is an ongoing epidemiological study set up in 1989 among employees of France’s national Gas and Electricity Company (EDF-GDF). The study uses an annual questionnaire to collect data on health, lifestyle, social and occupational factors. At inception, the cohort was constituted by 15,011 men and 5,614 women, aged 35–50 years old. During the first 17 years of follow-up (1989–2005), only 126 subjects (0.6%) were lost to follow-up. After a drop in the first 5 years of follow-up, almost 75% of participants returned the study questionnaire every year. As it was not always the same people who fail to respond each year, only 3.2% of the initial cohort never sent back any study questionnaire from 1989 to 2005 [13].

### Dietary Assessment

Dietary data were collected in 1998 through a 35-item qualitative Food Frequency Questionnaire (FFQ) for twenty food groups (never, 1–2 times/week or >2 times/week), eight items on low-fat food (yes/no), coffee consumption (number of cups per day) and six items on dietary behaviors like regular eating at breakfast, lunch and dinner and snacking between breakfast, lunch and dinner (never, rarely, often, every time). There was no portion size and nutrient intake assessment.

### Depressive Symptoms

Depressive symptoms were measured at several study phases in 1996, 1999, 2002, 2005 and 2008, using the CES-D scale [14]. This questionnaire includes 20 items that evaluate symptoms and behaviors characteristic of depressive disorder. Based on a previous French validation study, we used the following cut-off scores to define the presence of clinically significant depressive symptoms: ≥17 in men and 23 in women, out of 60 [15], [16]. Previous studies performed in the GAZEL cohort estimated the prevalence of high levels of depressive symptoms at 24.9% in men and 27.9% in women in 1996 and the prevalence at one year of major depressive episode at 7.6% in men and 17.9% in women [17].

### Covariates

Age was taken at the inception, in 1989. The measure of occupational position was taken from the employer’s records of grade of employment at age 35 for all participants as it was prior to the measure of alimentary consumption and represented mid-career status [18]. This measure has five levels, composed of executive, intermediate profession, employee, manual worker and missing at 35. Other covariates were measured at baseline in 1998, concurrently to the FFQ: body mass index (normal weight defined with a BMI <25 kg/m2, overweight with a BMI between 25 and 29 kg/m2 and obese with a BMI ≥30 kg/m2), marital status (single, divorced/separated/widowed or married/living with a partner), professional activity (active, on disability pension, retired, retired from EDF-GDF but still working), physical activity (at competition level, regularly, occasionally, no), smoking status (non-smokers, 1–10, 11–20 or 21 or more cigarettes/d), and alcohol consumption (none, 1–13, 14–27 or 28 or more units of alcohol/w in men; none, 1–6, 7–20, 21 or more units of alcohol/w).

### Statistical Analyses

#### Dietary patterns

**Figure 1:**
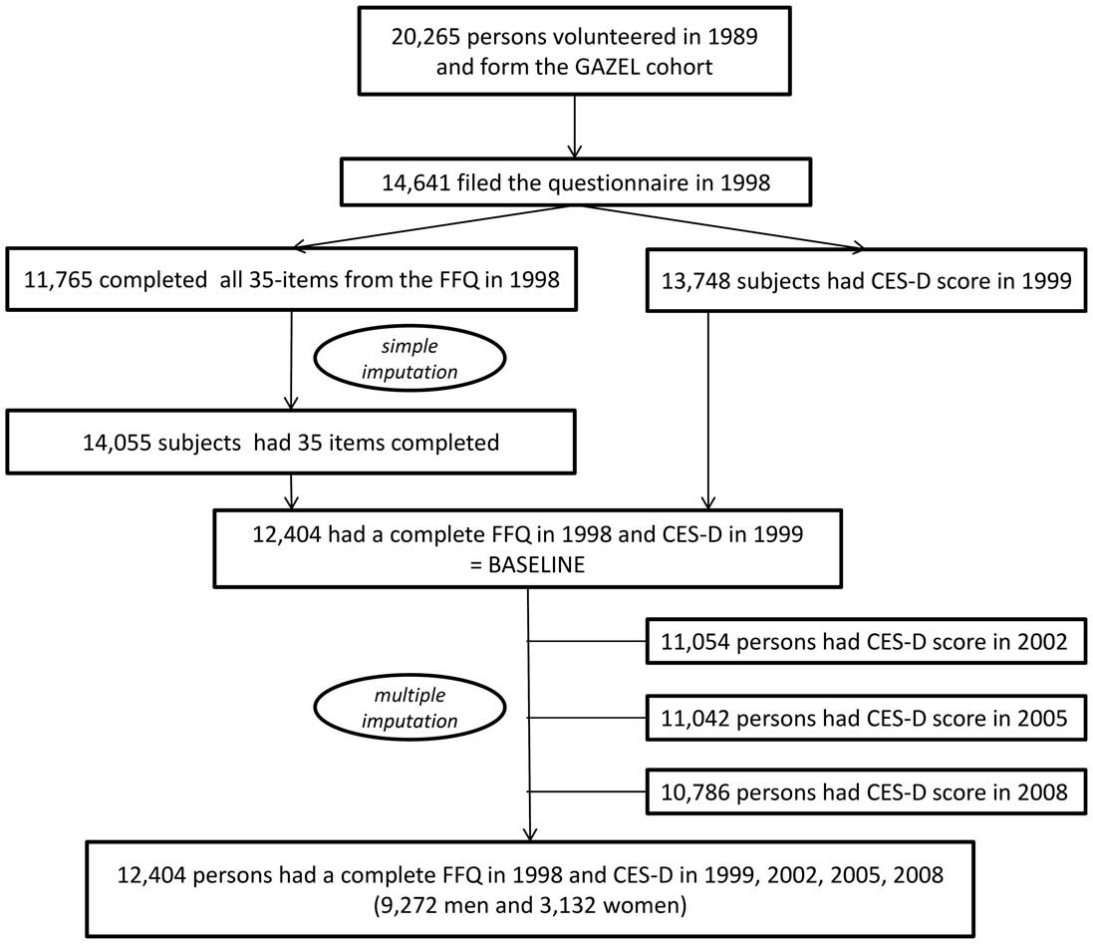
Flowchart describing the selection of participants in the study, Gazel cohort, 1989–2008.

All analyses were conducted separately for men and women and were restricted to subjects with available FFQ data in 1998 and CES-D data in 1999. Among the initial 20,625 subjects initially included in the cohort, 75% returned the 1998 study questionnaire, which served as the study baseline. Among them, 80.4% (11765/14641) completed 35 FFQ items (see Figure 1).We performed a simple imputation based on age, sex and occupational grade, to obtain complete dietary data for subjects with less than 5 missing observations. Finally, all 35 FFQ items were completed for 12,404 subjects who completed the CES-D in 1999, 84% of subjects having initially no missing data, 10% with one missing observation and 6% two to four missing observations. A principal component analysis (PCA) was used to assess dietary patterns in 1998. The patterns were rotated by orthogonal transformation (varimax rotation) to maintain uncorrelated factors and greater interpretability. We looked at the eigenvalues, investigated the scree plot to identify the eigenvalue drop, kept the first eight factors because there was no evidence of a two or three-factor solution and we took into account the face value of the patterns to make the final selection.

#### Treatment of missing data

Among subjects who completed the CESD in 1998 (baseline), only 13% (1618/12404) did not have a CES-D score at the end line in 2009. We performed Multiple Imputations by Chained Equation (MICE), which consists in imputing successively several values for each missing data item. Imputed data were CES-D in 1996, 2002, 2005 and 2008. The method of MICE assigns a new value for a given variable for every missing data. Because our aim was rather explicative than descriptive, we made the assumption of an “immortal cohort” [19], by allocating data after death using MICE. Five completed datasets, having the same characteristics as the observed data (variability and correlations) were generated, using linear regression to model the square root of CES-D [20], [21].The covariates used in the imputation models were the repeated CES-D and all the covariates previously described, adding physical and mental perceived health in 1998. Standard analyses were done separately on each completed dataset and then combined to obtain a global result (mi estimates, Stata v.11). Finally, 5 datasets were completed for 9,272 men and 3,132 women.

#### Analysis of longitudinal data

We performed all the analyses on the imputed data. To study the evolution of depression symptoms over time, according to dietary patterns in 1998, we used Generalized Estimating Equations (GEE) logistic regression for the analysis of CES-D as binary data, in order to take into account the correlation between repeated observations for the same subject [22]. We included initially all interactions terms between baseline dietary patterns and time to test for effect of baseline dietary pattern on change in depressive symptoms status. Interactions were non-significant so they were not kept in the final model. Coefficients and ORs (95CI) from these models can consequently be interpreted as averaged likelihood of being depressed over time (between 1999 and 2008). For each pattern, the GEE models included terms for pattern (in quartiles) and age in 1989, in order to obtain an averaged OR and 95CI of being depressed over time, adjusted for age (model 1). Then, the GEE models included terms for pattern, time (years), age in 1989, job position at 35 years old, marital status, physical activity, BMI, smoking status and alcohol consumption (model 2). Furthermore, to deal with reverse causality (dietary intakes modified by a pre-existing or latent depression), we performed the same analyses taking into account depression status in 1996 or 1999. The analyses were performed using Stata (version 11). For longitudinal modeling on imputed data, the mi estimates and xtgee procedures were used.

### Ethical Considerations

The GAZEL study received approval from the national commission overseeing ethical data collection in France (Commission Nationale Informatique et Liberté) and from the INSERM’s Institutional Review Board.

## Results

### Study Population

We selected for this analysis 12,404 subjects with complete data on diet patterns in 1998 and the CES-D in 1999 (9,272 men and 3,132 women). Among men, 1,979 subjects had up to three subsequent missing CES-D measures during follow-up and 794 among women. After multiple imputation (MICE), repeated CES-D were completed for all subjects. At baseline, men were on average 45.0 years old (SD = 2.9) and women 42.2 (4.2). In men, 52.2% were overweight and 9.9% were obese; in women, the corresponding figures were 22.7% and 7.6% respectively. The proportion of subjects still in professional activity was 68% in men and 79.2% in women. Among men, 17.1% were smokers and 15.7% were heavy alcohol consumers, while among women, they were 13.9% and 4.7% respectively.

### Dietary Patterns

Dietary patterns assessed in 1998 by principal component analysis are presented in Table 1. For each pattern, label was proposed based on food groups displaying factor loading >0.20. For men, on the first 8 patterns, we decide for simplicity of presentation to present 5: low-fat, healthy diet, western diet, fat-sweet and high snacking pattern. For women, we retained 6 dietary patterns: low-fat, healthy diet, traditional diet, animal protein pattern, high dessert and high snacking pattern. Other patterns as bread and butter pattern or dairy products pattern are not presented here because they were not found to be associated to symptoms of depression (results available upon request).

**Table 1:** Score coefficients related to dietary patterns derived from principal components analysis in men (n = 9272) and women (n = 3132) from the GAZEL cohort.

	MEN					WOMEN					
	Low-fatdiet	Healthydiet	Westerndiet	Fat-sweetpattern	Snacking	Low-fatdiet	Healthydiet	Traditionaldiet	Snacking	Animal protein pattern	Dessert
Meat			0.49							0.57	
Poultry										0.57	
Processed meat			0.38					−0.21		0.30	0.22
Fish								0.24			
Eggs										0.25	
Fried food			0.39								
Carbohydrates (starchy food)			0.29								
Cooked vegetables		0.42					0.36				
Raw vegetables		0.58					0.61				
Fruits		0.32	−0.23				0.28	0.24			
Dairy products											0.32
Fat-free dairy product	0.37					0.38					
Cheese											0.55
Fat-free cheese	0.55					0.50					
Desserts				0.45							0.41
Pastries				0.49							0.43
Oil		0.53					0.53				
Margarine	0.46					0.47					
Fat-free dishes	0.40					0.37					
High-sugar drinks				0.50				−0.21			
Diet sodas				−0.39		0.20					
Sugar free candies	0.36					0.27					
Coffee			0.25					−0.24			
Sweetener						0.23					0.23
Regular breakfast			−0.25					0.47			
Regular lunch								0.39		0.20	
Regular dinner								0.27			
Snacking in the morning					0.57				0.55		
Snacking in the afternoon					0.55				0.57		
Snacking in the evening					0.53				0.46		
% of variance explained	6.1	5.4	5.0	4.4	4.3	6.4	5.1	4.9	4.7	4.2	4.1

Factors loading lower than ±0.20 are not presented for simplicity. Only food items with factor loading higher than ±0.20 for all dietary patterns are presented in the table (butter, bread, sugar, milk and fat-free milk were omitted). For men, on the 8 first patterns, number 4 (milk and dairy product), number 5 (bread, cheese, butter, regular breakfast) and number 8 (regular breakfast, lunch and dinner) are no presented here because of low interpretability and absence of relation with symptoms of depression. For women, number 6 (milk and dairy product) and number 3 (bread, carbohydrates, butter, sugar) are no presented.

### Characteristics of the Population

The description of the population, according to quartiles of dietary patterns (Q1 and Q4), is presented in Table S1 and Table S2, for men and women respectively. The prevalence of subjects with depressive symptoms were 22.67%, 17.64%, 15.40% and 14.16% in men and 27.2%, 22.9%, 19.7% and 16.7% in women, in 1999, 2002, 2005 and 2008 respectively.

### Longitudinal Association between Dietary Patterns and Depression in Men

There were no significant interaction between dietary patterns and time in relation to depressive symptoms, suggesting therefore that baseline differences remained stable over time. Indeed, cross-sectional associations in 1999 and 2008 (adjusted for all covariates) are presented in Table 2.

**Table 2:** Odds-Ratio (95%CI) for probability of depressive symptoms in 1999 and 2008 for the upper quartile (Q4) (reference: 1^st^ quartile) of dietary patterns, in the GAZEL cohort.

Dietary pattern	OR 1999 (95%CI)	OR 2008 (95%CI)
**MEN**		
Low-fat	1.26 (1.10–1.45)	1.14 (0.97–1.34)
Healthy diet	0.69 (0.60–0.80)	0.73 (0.62–0.87)
Western diet	1.36 (1.18–1.57)	1.34 (1.13–1.60)
Fat-sweet	1.35 (1.17–1.55)	1.58 (1.31–1.90)
Snacking	1.49 (1.29–1.72)	1.58 (1.33–1.87)
**WOMEN**		
Low-fat	1.37 (1.09–1.73)	1.30 (0.95–1.78)
Healthy diet	0.80 (0.63–1.00)	0.72 (0.54–0.96)
Traditional diet	0.64 (0.51–0.82)	0.64 (0.47–0.86)
Snacking	1.51 (1.20–1.90)	1.59 (1.19–2.13)
Animal protein diet	0.91 (0.72–1.15)	0.96 (0.73–1.27)
Dessert	1.14 (0.91–1.43)	1.17 (0.90–1.53)

Table 3 shows longitudinal associations between dietary pattern scores categorized in quartiles in 1998 and depressive symptoms between 1999 and 2008. Participants from the highest quartiles of the healthy diet pattern at baseline were less likely to report subsequent depression than those from the lowest quartile, when adjusting for all the covariates (model 2): OR = 0.90, 95%CI (0.80–1.02), 0.78 (0.69–0.89) and 0.72 (0.63–0.83), p for trend <0.001. On the other hand, participants from the highest quartiles for the western diet, fat-sweet and high snacking pattern were at increased probability to report depression over the years (all p for trends <0.001). For the low-fat pattern, only the highest consumers (Q4) had an increased probability of depressive symptoms: OR = 1.16, 95%CI (1.02–1.31).

**Table 3:** Odds-ratios (95%CI) for probability of depressive symptoms by quartiles of dietary patterns in men of the GAZEL cohort (n = 9272).

Dietary pattern	Q1		Q2		Q3		Q4		P trend	
Low-fat										
Model 1	1		0.95 (0.84–1.09)		1.03 (0.91–1.17)		1.20 (1.06–1.36)			
Model 2	1		0.94 (0.82–1.07)		1.03 (0.91–1.17)		1.16 (1.02–1.31)		<0.01	
Healthy diet										
Model 1	1	0.84 (0.74–0.95)		0.72 (0.63–0.81)		0.66 (0.57–0.75)				
Model 2	1	0.90 (0.80–1.02)		0.78 (0.69–0.89)		0.72 (0.63–0.83)		<0.001		
Western diet										
Model 1	1		1.04 (0.91–1.19)		1.20 (1.05–1.37)		1.42 (1.25–1.61)			
Model 2	1		1.04 (0.91–1.18)		1.18 (1.04–1.35)		1.36 (1.19–1.54)		<0.001	
Fat-sweet										
Model 1	1		1.08 (0.95–1.23)		1.23 (1.08–1.40)		1.51 (1.31–1.71)			
Model 2	1		1.11 (0.97–1.27)		1.28 (1.12–1.45)		1.49 (1.30–1.71)		<0.001	
Snacking										
Model 1	1		1.00 (0.87–1.14)		1.19 (1.04–1.36)		1.50 (1.33–1.70)			
Model 2	1		1.02 (0.89–1.17)		1.23 (1.08–1.41)		1.50 (1.32–1.71)		<0.001	

Model 1 GEE model without interaction with time, adjusted for age in 1989.

Model 2 GEE model without interaction with time, adjusted for age in 1989, employment position at 35, professional activity, BMI, marital status, physical activity, tobacco smoking status and alcohol intake at baseline.

### Longitudinal Association between Dietary Patterns and Depressive Symptoms in Women

Cross-sectional associations for women in 1999 and 2008 (adjusted for all covariates) are presented in Table 2.

Table 4 shows longitudinal associations between dietary pattern scores categorized in quartiles in 1998 and depressive symptoms between1999and 2008. Women from the highest quartiles for the healthy pattern were less likely to report CES-D depression at baseline than those from the 1st quartile, respectively: OR = 0.88, 95%CI (0.73–1.07), 0.77 (0.63–0.95) and 0.75 (0.61–0.93), p for trend <0.001. For the traditional pattern, the protective effect was strong for all the quartiles of consumption compared to the reference (the lowest quartile): OR = 0.72, 95%CI (0.59–0.88), 0.76 (0.62–0.93) and 0.63 (0.50–0.80), p for trend <0.001.

**Table 4:** Odds-ratios (95%CI) for probability of depressive symptoms by quartiles of dietary patterns in women of the GAZEL cohort (n = 3132).

Dietary pattern	Q1		Q2		Q3		Q4		P trend	
Low-fat										
Model 1	1		0.87 (0.70–1.07)		1.04 (0.85–1.27)		1.42 (1.15–1.74)			
Model 2	1		0.88 (0.71–1.10)		1.05 (0.85–1.29)		1.39 (1.22–1.73)		<0.01	
Healthy diet										
Model 1	1	0.84 (0.69–1.01)		0.71 (0.58–0.87)		0.68 (0.55–0.84)				
Model 2	1	0.88 (0.73–1.07)		0.77 (0.63–0.95)		0.75 (0.61–0.93)		<0.001		
Traditional diet										
Model 1	1		0.68 (0.56–0.82)		0.70 (0.58–0.85)		0.58 (0.46–0.72)			
Model 2	1		0.72 (0.59–0.88)		0.76 (0.62–0.93)		0.63 (0.50–0.80)		<0.001	
Snacking										
Model 1	1		1.06 (0.86–1.30)		1.03 (0.82–1.28)		1.45 (1.18–1.78)			
Model 2	1		1.09 (0.89–1.35)		1.03 (0.82–1.28)		1.43 (1.16–1.76)		<0.01	
Animal protein diet										
Model 1	1		1.03 (0.84–1.25)		0.99 (0.80–1.21)		1.02 (0.84–1.24)			
Model 2	1		1.04 (0.85–1.27)		1.03 (0.84–1.27)		1.03 (0.84–1.26)		0.82	
Dessert										
Model 1	1		0.82 (0.66–1.02)		0.93 (0.76–1.15)		1.07 (0.88–1.30)			
Model 2	1		1.04 (0.85–1.27)		1.03 (0.84–1.27)		1.03 (0.84–1.26)		0.13	

Model 1 GEE model without interaction with time, adjusted for age in 1989.

Model 2 GEE model without interaction with time, adjusted for age in 1989, employment position at 35, professional activity, BMI, marital status, physical activity, tobacco smoking status and alcohol intake at baseline (and before if missing).

Regarding the patterns associated with an increased likelihood of depressive symptoms, only the low-fat and high snacking dietary patterns remained significantly associated with depressive symptoms at baseline in multivariate analysis in women from the highest quartile vs lowest: OR = 1.39, 95%CI (1.22–1.73) and OR = 1.43, 95%CI (1.16–1.76). The animal protein pattern and the dessert pattern were not significantly associated to an increased probability of depressive symptoms at baseline.

### Analysis Restricted to Subjects with Scores of CES-D<17/23, in Men and Women Respectively, in 1996 and 1999

To assess the possibility of reverse causality, we performed the same analysis among subjects presenting low levels of depressive symptoms simultaneously in 1996 and 1999 (data available upon request). Associations were non-significant for the healthy pattern, in men and women: OR = 1.00, 95%CI (0.78–1.28) for men and OR = 1.07, 95%CI (0.75–1.55) for women. However, for all other patterns, the associations remained unchanged in both men and women (including traditional diet for women).

## Discussion

To our knowledge, this is the first study to examine longitudinal associations between dietary patterns and repeated depressive symptoms assessment over 10 years. Several dietary patterns have been found to be associated with depressive symptoms in both cross sectional and longitudinal analyses.

In men, the western diet was significantly associated with an increased probability of depressive symptoms. A similar result was also found with three other patterns: high snacking, high consumption of low-fat food and fat-sweet food. In women, high snacking habits and high consumption of low-fat foods were associated with an elevated probability of depressive symptoms. Conversely, a traditional pattern based on fish and fruit consumption and regularity of the meals was associated with a reduced probability of depressive symptoms. These relationships demonstrated dose-response patterns and remained robust after adjustment for a wide range of potential confounding factors. Furthermore, since all interaction terms between dietary patterns and time in relation to depressive symptoms were not significant, these associations observed at baseline remained stable over time.

These results are concordant with other studies who demonstrated that a healthy pattern including fish consumption (like women’s traditional pattern in our study) was associated with a reduced probability and a western diet with an increased probability of depressive symptoms [4], [23]. An emergent idea is that processed food diet as in westernized diets could be related to inflammation and cardiovascular diseases, which are both related to the likelihood of depression [7], [24], [25]. Conversely, a healthy pattern rich in omega-3 fatty acids intakes has been linked with mood regulation [26]. Omega-3 fatty acids have anti-inflammatory properties and contribute to brain functioning and serotonin neurotransmission (e.g. providing fluidity to neurons cell membrane) [27]. Furthermore, there is evidence that adding omega-3 fatty acids to antidepressants may improve mood in major depression [28], [29].

Although the associations between dietary pattern and depressive symptoms over time were maintained in subject with no depressive symptoms in 1999 and 1996, making reverse causality unlikely for all patterns except healthy pattern, this hypothesis cannot be formally ruled out. Indeed the prospective association between diet and depressive symptoms may result from some unmeasured confounding factors such as past history of depression or negative life events. Life stress may promote not only depressive mood but also unhealthy dietary patterns [30], [31], presumably through its impact on the brain reward system [32]. Unhealthy dietary pattern refers to eating behaviors like snacking or craving for specific food. Much of the available evidence on food cravings focused on the connection between diet and mood. A frequently proposed theory is that food is being used to ameliorate unpleasant affective states, through the increase of serotonin, a brain neurotransmitter. Women in particular report extreme craving for foods that are both sweet and high in fat (e.g., candies, pastries or ice cream) [33], [34].

In both sexes, dietary patterns characterized by healthier diets (raw and cooked vegetables, oil and fruits) were associated with reduced probabilities of depressive symptoms. However, the protective effect observed for this pattern was reduced towards the null when the analyses were restricted to participants without elevated depressive symptoms in 1996 or 1999. This suggests the existence of possible reverse causality; depressed participants being less likely to consume healthy food (raw and cooked vegetables). Finally, subjects with the highest consumption of low-fat food could have the poorest health (like important rates of obesity), suggesting a possible selection bias.

Our results need to be interpreted in light of several limitations. First, the GAZEL cohort includes individuals who, at the time they were recruited into the cohort, held a stable job and volunteered to participate in an epidemiological cohort. Thus, one might expect that the frequency of depressive symptoms could be lower than in the general population. However, past research in this cohort showed that depressive symptoms are in fact as frequent in the GAZEL cohort as in the French population [35]. Second, depression in our study was assessed using the CES-D, which does not allow a clinical diagnosis of major depression [36]. However, this is a well-established instrument for identifying depressive symptoms, which has been shown to be reliable and valid across varied cultural and sociodemographic settings. Third, there was no portion size and nutrient intake was not assessed given that our food questionnaire asked only the weekly frequency of consumption of several items and the dietary questionnaire was not validated for the assessment of dietary patterns. However, the main aim of the study was to examine the magnitude of the associations between dietary patterns and symptoms of depression. An additional limitation would be that we made two strong assumptions: i) there are gender differences in depressive symptomatology and dietary behaviors, ii) all the data (dietary and lifestyle habits) collected at baseline are stable over time. However, dietary behaviors are different across gender, as depressive symptomatology [37]–[40]. Moreover, our aim was to study the predictive value of dietary patterns assessed at one precise moment, over the ten following year’s period. Finally, concerning missing data, imputation models could be questionable because CES-D values could depend on other variables that were not included in the imputations models or that missing data would not be missing at random. For missing data caused by death, the choice of imputation models is even more questionable. However, mortality being not excessive (3.7% in men and 1.8% in women), we estimated that it was negligible.

On the other hand, this study has several important strengths, including the large sample size comprising subjects from both genders, the longitudinal design with repeated measures for depression over a long period of time and the inclusion of a wide range of potential confounding variables.

### Conclusion

This is the first prospective study, using repeated CES-D measures, showing that several dietary patterns are predictive of the occurrence of depressive symptoms. In particular, we identified a traditional pattern characterized by fruits and fish consumption and regularity of the meals in women and unhealthy patterns in both sexes, as western diet, animal protein pattern, low-fat diet, high snack diet, and high dessert and fat-sweet diet. In both sexes, a healthy pattern characterized by vegetables consumption was found to be associated with a lower likelihood of depressive symptoms but there was probably a reverse causality effect for this pattern. Finally, associations between high quartiles of healthy and unhealthy patterns and symptoms of depression remain surprisingly stable over a period of 10 years.

## Supporting Information

Table S1 Baseline characteristics in the lowest (Q1) and upper (Q4) quartiles of dietary patterns in 9262 men from the GAZEL cohort
(DOCX)

Table S2 Sample characteristics for the lowest (Q1) and highest (Q4) quartiles of 6 food patterns identified at baseline for 3132 women from the GAZEL cohort
(DOCX)
