# Supplementary material for: Association between Dietary Patterns and Depressive Symptoms Over Time: A 10-Year Follow-Up Study of the GAZEL Cohort
Source: PLoS One. 2012 Dec 12;7(12):e51593. doi: 10.1371/journal.pone.0051593 (PMC3520961; doi:10.1371/journal.pone.0051593)
Supplement: Table S1 — Baseline characteristics in the lowest (Q1) and upper (Q4) quartiles of dietary patterns in 9262 men from the GAZEL cohort (DOCX) [file pone.0051593.s001.docx]

**Table S1**: Baseline characteristics in the lowest (Q1) and upper (Q4) quartiles of dietary patterns in 9262 men from the GAZEL cohort.

| **Sample characteristics** % (SD) | | **Low-fat diet** | | **Healthy diet** | | **Western diet** | | **Fat-sweet** | | **Snacking** | |
| --- | --- | --- | --- | --- | --- | --- | --- | --- | --- | --- | --- |
|  | | **Q1** | **Q4** | **Q1** | **Q4** | **Q1** | **Q4** | **Q1** | **Q4** | **Q1** | **Q4** |
| Age in 1989 (y) mean (range) | | | | | | | | | | | |
|  | | 44.9 (0.3) | 45.1 (0.3) | 44.6 (0.2) | 45.4 (0.3) | 45.4 (0.3) | 44.6 (0.3) | 45.4 (0.3) | 44.6 (0.3) | 45.1 (0.3) | 44.9 (0.3) |
| BMI (kg/m^2^) | |  |  |  |  |  |  |  |  |  |  |
|  | <25 | 42.9 (0.4) | 33.4 (0.4) | 34.5 (0.4) | 41.1 (0.4) | 41.1 (0.4) | 36.4 (0.4) | 35.4 (0.4) | 38.6 (0.4) | 35.7 (0.4) | 39.8 (0.4) |
|  | 25 - 29.9 | 48.7 (0.5) | 54.1 (0.5) | 53.4 (0.5) | 50.7 (0.5) | 51.5 (0.5) | 51.1 (0.4) | 53.5 (0.5) | 50.8 (0.5) | 53.7 (0.5) | 50.1 (0.5) |
|  | ≥30 | 8.4 (0.2) | 12.4 (0.3) | 12.0 (0.3) | 8.2 (0.2) | 7.4 (0.2) | 12.5 (0.3) | 11.1 (0.3) | 10.6 (0.3) | 10.6 (0.3) | 10.1 (0.3) |
| Professional activity | | | | | | | | | | | |
|  | Yes | 69.6 (4.3) | 65.8 (0.4) | 74.2 (0.4) | 63.0 (0.4) | 63.7 (0.4) | 74.2 (0.4) | 64.1 (0.4) | 73.3 (0.4) | 69.5 (0.4) | 66.8 (0.4) |
|  | Long disease | 0.5 (0.1) | 0.8 (0.1) | 0.9 (0.1) | 0.3 (0.1) | 69 (0.1) | 69 (0.1) | 0.8 (0.1) | 0.9 (0.1) | 0.9 (0.1) | 0.7 (0.1) |
|  | Retired | 28.6 (4.2) | 31.6 (0.4) | 23.8 (0.4) | 35.5 (0.4) | 34.3 (0.4) | 24 (0.4) | 33.5 (0.4) | 25.1 (0.4) | 28.1 (0.4) | 31 (0.4) |
|  | Retired still active | 1.3 (0.1) | 1.8 (0.1) | 1.1 (0.1) | 1.2 (0.1) | 1.3 (0.1) | 1.1 (0.1) | 1.5 (0.1) | 0.8 (0.1) | 1.4 (0.1) | 1.5 (0.1) |
| Employment position at 35 years old | | | | | | | | | | | |
|  | Executive | 19.8 (0.4) | 13.4 (0.3) | 15.0 (0.3) | 20.3 (0.4) | 18.3 (0.3) | 17.4 (0.3) | 16.4 (0.3) | 18.3 (0.3) | 20.5 (0.4) | 13.9 (0.3) |
|  | Intermediate profession | 58.0 (0.5) | 54.8 (0.5) | 59.5 (0.5) | 54.9 (0.5) | 56.9 (0.5) | 55.6 (0.5) | 59.2 (0.4) | 56.3 (0.5) | 56.9 (0.4) | 57.4 (0.4) |
|  | Employee | 4.4 (0.2) | 5.5 (0.2) | 5.9 (0.2) | 5.0 (0.2) | 5 (0.2) | 5.5 (0.2) | 4.6 (0.2) | 4.6 (0.2) | 4.9 (0.2) | 5.6 (0.2) |
|  | Manual worker | 14.9 (3.3) | 22.6 (0.3) | 17.1 (0.3) | 16.8 (0.3) | 17 (0.3) | 18.3 (0.3) | 16.7 (0.3) | 18.2 (0.3) | 14.8 (0.3) | 19.8 (0.4) |
|  | Missing at 35 | 2.9 (0.1) | 3.7 (0.2) | 2.9 (0.1) | 3.1 (0.2) | 2.8 (0.1) | 3.1 (0.2) | 3.1 (0.2) | 2.6 (0.1) | 2.8 (0.1) | 3.3 (0.2) |
| Marital status | | | | | | | | | | | |
|  | Single | 2.0 (0.1) | 2.4 (0.1) | 2.8 (0.1) | 1.6 (0.1) | 2.2 (0.1) | 2.1 (0.1) | 2.1 (0.1) | 3.1 (0.2) | 2.3 (0.1) | 2.1 (0.1) |
|  | In couple^a^ | 91.9 (0.2) | 91.5 (0.2) | 88.3 (3.0) | 94.8 (0.2) | 92.1 (0.2) | 92.3 (0.2) | 92.4 (0.2) | 89.5 (0.3) | 91.9 (0.2) | 91.5 (0.2) |
|  | Separated^b^ | 6.1 (0.2) | 6.1 (0.2) | 8.9 (0.3) | 3.5 (0.2) | 5.6 (0.2) | 5.6 (0.2) | 5.5 (0.2) | 7.4 (0.2) | 5.8 (0.2) | 6.4 (0.2) |

|  |  | |  | |  | |  | |  |  |  |
| --- | --- | --- | --- | --- | --- | --- | --- | --- | --- | --- | --- |
| **Sample characteristics** % (SD) | | **Low-fat diet** | | **Healthy diet** | | **Western diet** | | **Fat-sweet** | | **Snacking** | |
|  |  | **Q1** | **Q4** | **Q1** | **Q4** | **Q1** | **Q4** | **Q1** | **Q4** | **Q1** | **Q4** |
| Physical activity | | | | | | | | | | | |
| Competition | 6.6 (0.2) | 6.4 (0.2) | 6.3 (0.2) | 5.7 (0.2) | 7.3 (0.2) | 5.7 (0.2) | 5.5 (0.2) | 6.9 (0.2) | 5.6 (0.2) | 6.8 (0.2) |  |
| Regularly | 29.5 (0.4) | 28.2 (0.4) | 24.6 (0.4) | 32.1 (0.4) | 35.2 (0.4) | 22.4 (0.4) | 29.8 (0.5) | 27.8 (0.4) | 28.5 (0.4) | 29.1 (0.4) |  |
| Occasionally | 29.7 (0.4) | 28.6 (0.4) | 27.7 (0.4) | 28.3 (4.2) | 27.3 (0.4) | 29.1 (0.4) | 26.8 (0.4) | 29.8 (0.4) | 27.8 (0.4) | 28.4 (0.4) |  |
| No | 34.2 (0.5) | 36.8 (0.5) | 41.4 (0.5) | 34.0 (0.4) | 30.1 (0.4) | 42.8 (0.5) | 37.9 (0.4) | 35.5 (0.4) | 38.1 (0.4) | 35.9 (0.4) |  |
| Tobacco | | | | | | | | | | | |
|  | Non smoker | 83.7 (0.3) | 83.9 (0.3) | 77.3 (0.4) | 84.6 (0.3) | 88.6 (0.3) | 76.3 (0.4) | 82.1 (0.3) | 82.8 (0.3) | 80.8 (0.4) | 84.1 (0.3) |
|  | Smoker | 16.3 (0.3) | 16.0 (0.3) | 22.7 (0.4) | 15.3 (0.3) | 11.4 (0.3) | 23.6 (0.4) | 17.9 (0.3) | 17.2 (0.3) | 19.2 (0.4) | 15.9 (0.3) |
| Alcohol | | | | | | | | | | | |
|  | Abstinent | 7.9 (0.2) | 9.1 (0.3) | 7.9 (0.2) | 7.7 (0.2) | 11.6 (0.3) | 5.7 (0.2) | 7.5 (0.2) | 9.9 (0.3) | 5.9 (0.2) | 10.7 (0.3) |
|  | Small consumer | 51.5 (0.5) | 52.0 (0.5) | 51.5 (0.5) | 51.5 (0.5) | 57.7 (0.5) | 42.5 (0.5) | 49.5 (0.5) | 52.7 (0.5) | 43.5 (0.5) | 55.1 (0.5) |
|  | High consumer | 40.5 (0.4) | 38.8 (0.4) | 40.6 (0.4) | 40.8 (0.4) | 30.6 (0.4) | 51.7 (0.5) | 43.0 (0.5) | 37.4 (0.4) | 50.5 (0.5) | 34.2 (0.4) |
|  |  |  |  |  |  |  |  |  |  |  |  |
| CESD ≥ 17^c^ (%) | | | | | | | | | | | |
|  | 1999 | 21.1 (0.8) | 25.3 (0.9) | 28.2 (0.9) | 19.1 (0.8) | 20.0 (0.8) | 27.2 (0.9) | 19.6 (0.8) | 25.8 (0.9) | 20.1 (0.8) | 27.0 (0.9) |
|  | 2002 | 16.8 (0.8) | 20.4 (0.9) | 21.4 (0.9) | 15.2 (0.8) | 15.7 (0.8) | 21.3 (0.9) | 15.9 (0.8) | 21.1 (0.9) | 16.4 (0.8) | 21.6 (0.9) |
|  | 2005 | 15.6 (0.8) | 17.5 (0.8) | 19.1 (0.9) | 13.3 (0.7) | 14.2 (0.7) | 18.5 (0.8) | 13.2 (0.8) | 18.9 (0.8) | 13.3 (0.7) | 19.4 (0.9) |
|  | 2008 | 14.5 (0.7) | 16.7 (0.8) | 17.7 (0.9) | 12.8 (0.7) | 13.2 (0.7) | 17.5 (0.8) | 12.6 (0.7) | 18.5 (0.9) | 12.9 (0.7) | 18.9 (0.8) |

*Food patterns were selected after principal component analysis. All analysis was performed on imputed datasets.*

*^a^ married or living with a partner*

*^b^ separated, divorced or widowed*

*^c^ Center for Epidemiologic Studies Depression Scales (CES-D)*
