# Supplementary material for: Association between Dietary Patterns and Depressive Symptoms Over Time: A 10-Year Follow-Up Study of the GAZEL Cohort
Source: PLoS One. 2012 Dec 12;7(12):e51593. doi: 10.1371/journal.pone.0051593 (PMC3520961; doi:10.1371/journal.pone.0051593)
Supplement: Table S2 — Sample characteristics for the lowest (Q1) and highest (Q4) quartiles of 6 food patterns identified at baseline for 3132 women from the GAZEL cohort (DOCX) [file pone.0051593.s002.docx]

**Table S2**: Sample characteristics for the lowest (Q1) and highest (Q4) quartiles of 6 food patterns identified at baseline for 3132 women from the GAZEL cohort

|  |  | **Low-fat diet** | | **Healthy diet** | | **Traditional diet** | | **Western diet** | | **Dessert and sweets** | | **Snacking** | |
| --- | --- | --- | --- | --- | --- | --- | --- | --- | --- | --- | --- | --- | --- |
| **Sample characteristics** | | **Q1** | **Q4** | **Q1** | **Q4** | **Q1** | **Q4** | **Q1** | **Q4** | **Q1** | **Q4** | **Q1** | **Q4** |
| Age in 1989 (y) mean (range) | | | | | | | | | | | | | |
|  |  | 42.1 (0.1) | 42.2 (0.1) | 41.6 (0.1) | 42.9 (0.1) | 41.2 (0.1) | 42.9 (0.1) | 42.9 (0.1) | 41.5 (0.1) | 42.4 (0.1) | 41.7 (0.1) | 42 (0.1) | 41.8 (0.1) |
| BMI (kg/m^2^) | | | | | | | | | | | | | |
|  | <25 | 77.1 (0.7) | 58.7 (0.8) | 70.8 (0.7) | 70.5 (0.7) | 71.3 (0.7) | 69.8 (0.7) | 71 (0.7) | 67 (0.7) | 75.5 (0.7) | 64.6 (0.8) | 65.9 (0.7) | 70.6 (0.7) |
|  | 25 - 29.9 | 17.7 (0.6) | 28.9 (0.7) | 21.7 (0.6) | 21.3 (0.6) | 21.2 (0.6) | 22.9 (0.7) | 21.6 (0.6) | 25.3 (0.7) | 18.9 (0.6) | 26.3 (0.7) | 24.8 (0.7) | 22.8 (0.7) |
|  | ≥30 | 5.1 (0.3) | 12.4 (0.5) | 7.4 (0.4) | 8.2 (0.4) | 7.4 (0.4) | 7.3 (0.4) | 7.4 (0.4) | 7.7 (0.4) | 5.6 (0.4) | 9.1 (0.4) | 9.3 (0.5) | 6.6 (0.4) |
| Professional activity | | | | | | | | | | | | | |
|  | Yes | 79.7 (0.6) | 78.7 (0.6) | 84.5 (0.6) | 73.8 (0.7) | 87.6 (0.5) | 72.3 (0.7) | 77.8 (0.7) | 82.1 (0.6) | 83.4 (0.6) | 79.6 (0.6) | 81.5 (0.6) | 80.6 (0.6) |
|  | Long disease | 1.7 (0.2) | 2.7 (0.2) | 1.9 (0.2) | 1.5 (0.2) | 1.9 (0.2) | 1.8 (0.2) | 1.8 (0.2) | 2.2 (0.2) | 1.3 (0.2) | 1.5 (0.2) | 2.4 (0.2) | 1 (0.2) |
|  | Retired | 17.5 (0.6) | 18 (0.6) | 12.3 (0.5) | 23.7 (0.7) | 9.7 (0.5) | 24.8 (0.7) | 19.8 (0.6) | 14.8 (0.6) | 14.7 (0.6) | 17.6 (0.6) | 15.4 (0.6) | 17.8 (0.6) |
|  | Retired still active | 1.1 (0.2) | 0.6 (0.1) | 1.3 (0.2) | 0.9 (0.1) | 0.8 (0.1) | 1.1 (0.2) | 0.6 (0.1) | 0.9 (0.1) | 0.6 (0.1) | 1.3 (0.2) | 6.3 (0.1) | 0.6 (0.1) |
| Employment position | | | | | | | | | | | | | |
|  | Executive | 6.7 (0.4) | 6.2 (0.4) | 6.6 (0.4) | 6.9 (0.4) | 7.4 (0.4) | 5.5 (3.6) | 8.7 (0.4) | 4.1 (0.3) | 5.9 (0.4) | 7.1 (0.4) | 8.3 (0.4) | 5.7 (0.4) |
|  | Intermediate profession | 52.5 (0.8) | 49.9 (0.8) | 49.9 (0.8) | 51.7 (0.8) | 53 (0.8) | 51.3 (0.8) | 55 (0.8) | 48 (0.8) | 49.4 (0.8) | 51.1 (0.8) | 54 (0.8) | 51.3 (0.8) |
|  | Employee | 34.1 (7.6) | 37. (0.8) | 37.4 (0.8) | 35.2 (0.8) | 33.1 (0.7) | 36.1 (0.8) | 31 (0.7) | 40.3 (0.8) | 37.4 (0.8) | 36.6 (0.8) | 31.8 (0.7) | 36.8 (0.8) |
|  | Manual worker | 0.8 (0.1) | 0.6 (0.1) | 0.7 (0.1) | 1.0 (0.2) | 0.6 (0.1) | 0.5 (0.1) | 0.3 (0.1) | 1.3 (0.2) | 1.0 (0.2) | 0.9 (0.1) | 0.2 (0.1) | 0.9 (0.1) |
|  | Missing at 35 | 5.9 (0.4) | 6.1 (0.4) | 5.2 (0.3) | 5.1 (0.3) | 5.9 (0.4) | 6.5 (0.4) | 5.0 (0.3) | 6.2 (0.4) | 6.2 (0.4) | 4.2 (0.3) | 5.6 (0.4) | 5.2 (0.4) |
| Marital status | | | | | | | | | | | | | |
|  | Single | 7.3 (0.4) | 6.9 (0.4) | 6.8 (0.4) | 5.1 (0.3) | 5.9 (0.4) | 6.2 (0.4) | 5.2 (0.3) | 6 (0.4) | 8.8 (0.4) | 5.5 (0.4) | 6.8 (0.4) | 6.6 (0.4) |
|  | In couple^a^ | 74.4 (0.7) | 72.3 (0.7) | 67.7 (0.7) | 80.4 (0.6) | 69.2 (0.7) | 77.3 (0.7) | 76.4 (0.7) | 72 (0.7) | 65.1 (0.8) | 78.7 (0.6) | 71.6 (0.7) | 76 (0.7) |
|  | Separated^b^ | 18.3 (0.6) | 20.8 (0.6) | 25.5 (0.7) | 14.4 (0.6) | 24.9 (0.7) | 16.5 (0.6) | 18.4 (0.6) | 22 (0.7) | 26 (0.7) | 15.8 (0.6) | 21.6 (0.6) | 17.4 (0.6) |

|  |  | **Low-fat diet** | | **Healthy diet** | | **Traditional diet** | | **Western diet** | | **Dessert and sweets** | | **Snacking** | |
| --- | --- | --- | --- | --- | --- | --- | --- | --- | --- | --- | --- | --- | --- |
| **Sample characteristics** | | **Q1** | **Q4** | **Q1** | **Q4** | **Q1** | **Q4** | **Q1** | **Q4** | **Q1** | **Q4** | **Q1** | **Q4** |
| Physical activity | | | | | | | | | | | | | |
|  | Competition/ regularly | 35.4 (0.8) | 35.1 (0.8) | 31.3 (0.7) | 38.6 (0.8) | 27.8 (0.7) | 44.2 (0.8) | 32.9 (0.8) | 36.7 (0.8) | 40.5 (0.8) | 30 (0.7) | 34.8 (0.8) | 36.7 (0.8) |
|  | Occasionally | 21.4 (0.7) | 23.7 (0.7) | 18.6 (0.6) | 24.5 (0.7) | 23.4 (0.7) | 21.5 (0.7) | 22 (0.7) | 21.7 (0.7) | 20.7 (0.7) | 24.1 (0.7) | 19.7 (0.7) | 21.2 (0.7) |
|  | No | 43.1 (0.8) | 41.2 (0.8) | 50.1 (0.8) | 36.8 (0.8) | 48.7 (0.8) | 34.3 (0.8) | 45.1 (0.8) | 41.6 (0.8) | 38.8 (0.8) | 46 (0.8) | 45.4 (0.8) | 42 (0.8) |
| Tobacco | | | | | | | | | | | | | |
|  | Non smoker | 87.0 (0.5) | 86.5 (0.5) | 81.7 (0.6) | 88.3 (0.5) | 77.6 (0.7) | 92.08 (0.4) | 82.5 (0.6) | 88.4 (0.5) | 86.3 (0.5) | 84.5 (0.6) | 82.2 (0.6) | 89.9 (0.5) |
|  | Smoker | 13.0 (0.5) | 13.5 (0.5) | 18.3 (0.6) | 11.7 (0.5) | 22.3 (0.7) | 7.9 (0.4) | 17.5 (0.6) | 11.6 (0.5) | 13.7 (0.5) | 15.4 (0.6) | 17.8 (0.6) | 10.1 (0.5) |
| Alcohol | | | | | | | | | | | | | |
|  | Abstinent | 19.4 (0.6) | 26.4 (0.7) | 25.0 (0.7) | 20.9 (0.6) | 19.4 (0.6) | 24.9 (0.7) | 20.2 (0.6) | 26.7 (0.7) | 26.3 (0.7) | 19.5 (0.6) | 28 (0.7) | 20.4 (0.6) |
|  | Small intake | 48.6 (0.8) | 50.4 (0.8) | 48.3 (0.8) | 49.4 (0.8) | 47.2 (0.8) | 52.7 (0.8) | 43.5 (0.8) | 53.6 (0.8) | 53.6 (0.8) | 47.2 (0.8) | 48.4 (0.8) | 59.8 (0.8) |
|  | High consumer | 31.9 (0.7) | 23.1 (0.7) | 26.7 (0.7) | 29.6 (0.7) | 33.3 (0.7) | 22.3 (0.7) | 36.3 (0.8) | 19.7 (0.6) | 20.0 (0.6) | 33.2 (0.7) | 23.6 (0.7) | 29.7 (0.7) |
| CES-D ≥ 23^c^ (%) | | | | | | | | | | | | | |
|  | 1999 | 25.5 (1.5) | 32.3 (1.7) | 31.3 (1.6) | 23.7 (1.5) | 34.2 (1.7) | 25.0 (1.5) | 24.3 (1.5) | 33.1 (1.7) | 28.1 (1.6) | 25.5 (1.6) | 28.0 (1.6) | 28.9 (1.6) |
| 2002 | 21.2 (1.6) | 27.8 (1.8) | 26.7 (1.7) | 20.0 (1.5) | 29.2 (1.9) | 18.0 (1.4) | 19.9 (1.5) | 28.3 (1.7) | 23.9 (1.6) | 22.1 (1.5) | 22.8 (1.7) | 25.2 (1.7) |  |
|  | 2005 | 18.9 (1.4) | 25.6 (1.6) | 24.0 (1.6) | 16.8 (1.5) | 25.2 (1.5) | 16.7 (1.6) | 18.5 (1.4) | 23.6 (1.5) | 18.0 (1.4) | 21.5 (1.5) | 21.4 (1.6) | 21.2 (1.5) |
|  | 2008 | 16.4 (1.4) | 20.3 (1.6) | 20.7 (1.5) | 14.5 (1.3) | 22.3 (1.6) | 13.9 (1.3) | 14.9 (1.3) | 21.3 (1.5) | 16.9 (1.4) | 17.2 (1.4) | 17.8 (1.4) | 19.4 (1.4) |

*Food patterns were selected after principal component analysis. All analysis was performed on imputed datasets.*

*^a^ Married or living with a partner*

*^b^ Separated, divorced or widowed*

*^c^ Center for Epidemiologic Studies Depression Scales (CES-D)*
